# xCELLigence™ RTCA system in cancer biology: innovations in real-time functional profiling

**DOI:** 10.1007/s10616-026-01000-z

**Published:** 2026-05-26

**Authors:** Charlene Andraos, Jitcy Saji Joseph, Wells Utembe, Millicent Magogotya, Olumayowa Oriyomi, Oladapo Fagbohun

**Affiliations:** 1 Department of Toxicology and Biochemistry, A division of National Health Laboratory Services, National Institute for Occupational Health, Johannesburg, South Africa; 2 Water Research Unit Group, North West University, Potchefstroom, 2520 South Africa; 3 Department of Life & Consumer Sciences, College of Agriculture and Environmental Sciences, University of South Africa, Johannesburg, 1709 South Africa; 4 Environmental Health Division, School of Public Health and Family Medicine, University of Cape Town, Cape Town, 7925 South Africa; 5 School of Engineering, University of Greater Manchester, Manchester, BL3 5AB UK; 6 Department of Biology, Wilmington College, 1870 Quaker Way, Wilmington, OH 45177 USA

**Keywords:** xCELLigence RTCA, Impedance-based technology, Cell migration, Cell Index, Precision oncology

## Abstract

The molecular mechanisms and interactions for the complex cancer pathogenesis are still poorly understood. Traditional and conventional endpoint migration assays such as MTT/XTT/CCK-8, transwell migration, clonogenic, and false positives/negatives assays, do not capture the kinetics of the 3D tumor microenvironment. Therefore, there is the need for high-resolution kinetic data on cancer cell invasion, proliferation, migration, cytotoxicity, adhesion, and differentiation, to understand transient and adaptive responses that are often missed by static measurements for highly effective cancer immunotherapeutics. Here, we review the xCELLigence™ Real-Time Cell Analysis (RTCA) system that represents a major advancement in cancer research by enabling non-invasive, label-free, and continuous monitoring of cellular behaviour through impedance-based technology. Over the years, and particularly since 2020, RTCA has been increasingly applied and relied upon across diverse areas of cancer research, including kinase inhibition, gene silencing, metabolic targeting, immunotherapy, nanomedicine, and three-dimensional tumour models. We first analyse the recent advances in the applications of xCELLigence™ RTCA, with a specific emphasis on how impedance-derived kinetic signatures enhance mechanistic understanding of tumor cell behaviour. We then critically discuss quantitative metrics derived from real-time impedance measurements, compare RTCA with conventional endpoint assays, and highlight its translational relevance for drug development and precision oncology. Finally, we outline current limitations and emerging directions that may further consolidate RTCA as a predictive and integrative platform in preclinical cancer research.

**Graphical abstract:**

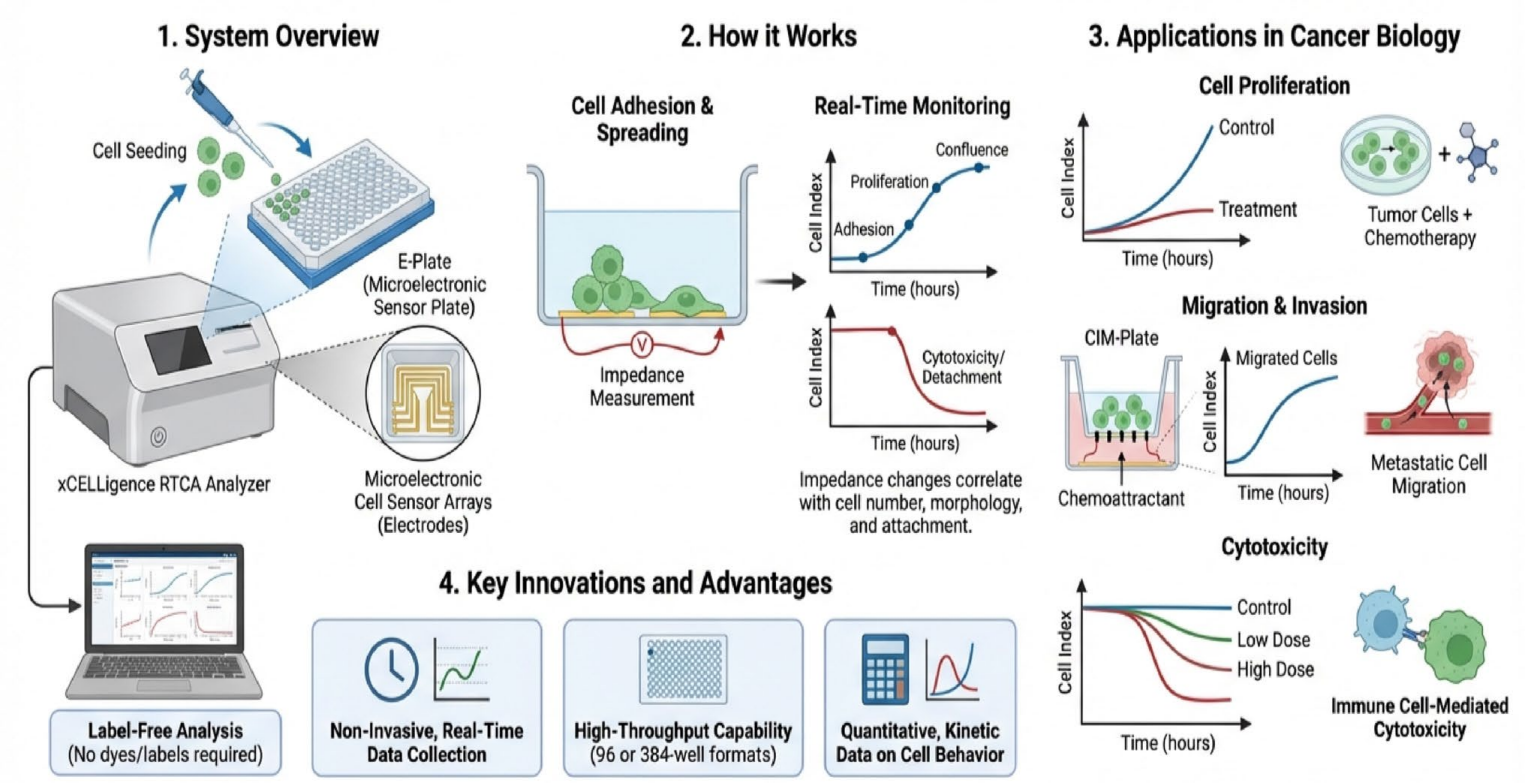

## Introduction

Cancer progression and therapeutic resistance are governed by highly dynamic cellular processes that evolve in response to genetic, epigenetic, metabolic, and microenvironmental regulatory factors (Marei 2025; Song et al. 2025). Traditional in vitro assays used in cancer research, including MTT, flow cytometry, clonogenic survival, or endpoint migration assays, typically provide static snapshots of cellular responses at predefined time points, which are irreversible, time-consuming, and do not reflect the 3D tumor microenvironment (Forgie et al. 2024; Pijuan et al. 2019). While informative, these approaches frequently obscure transient, delayed, or adaptive cellular behaviors that are critical for understanding treatment efficacy and resistance mechanisms (Khalef et al. 2024). Therefore, since these analyses do not capture the complex, time- and dose-dependent kinetics of cancer cell behavior, there is a need for a live-cell imaging technique that can capture cell behavior in non-invasive, time-dependent, and high-throughput real-time (Creighton 2023). To overcome these limitations, modern studies are shifting toward or adopting Real-Time Cell Analysis (RTCA) (Stefanowicz-Hajduk and Ochocka 2020), which enables non-invasive, continuous monitoring of transient events in cells, such as apoptosis and cancer cell migration (Alieva et al. 2023).

The xCELLigence™ Real-Time Cell Analyzer (RTCA) system, also known as RT-CES™ system, originally developed by ACEA Biosciences and co-developed by Roche and ACEA, and now commercialized by Agilent Technologies, is an impedance-based, label-free platform that enables continuous, real-time quantification of cellular behaviour and dynamic biological processes (Hamidi et al. 2017; Khan et al. 2025). The system relies on a gold microelectrode biosensor built into E-plate™ microtiter plates (Cerignoli et al. 2018). The sensor measures changes in the electrical impedance of cell populations in the microelectrode-coated well plates (Sagot et al. 2023) from cell attachment, growth, or morphological shifts or functional changes. Other plates included in the RTCA system are the CIM and CardioECR plates, which are specific in nature (Stefanowicz-Hajduk and Ochocka 2020). While the E-Plate assesses cell proliferation, adhesion, and cytotoxicity, the CIM-Plate specializes in migration and invasion via its micro-porous membrane and the CardioECR Plate is used specifically for cardiomyocyte function, including electrical activity (Stefanowicz-Hajduk and Ochocka 2020; Türker Şener et al. 2017; Yan et al. 2018a, b). Ultimately, the system converts electric impedance measurements into a dimensionless parameter known as the Cell Index (CI), which quantitatively reflects cell number, viability, morphology, and adhesion strength, providing a robust metric for real-time analysis (Reitinger et al. 2012). By measuring changes in electrical impedance generated by cells interacting with gold microelectrode-coated plates, RTCA enables continuous, quantitative assessment of cell proliferation, viability, morphology, adhesion, migration, and invasion (Türker Şener et al. 2017). The resulting kinetic profiles (CI) provide a dynamic cellular fingerprint that reflects both immediate and long-term responses to experimental perturbations (Maiti et al. 2024), thereby allowing researchers to pinpoint the precise moment a cellular change occurs (Hamidi et al. 2017; Khan et al. 2025; Kociubiński 2021; Lebourgeois et al. 2018).

Since its introduction, the RTCA platform has been adopted across a wide range of biomedical disciplines and research areas such as drug development, cancer research, immunology, toxicology, virology, stem cell research, neurobiology, tissue engineering, and regenerative medicine, with particular emphasis in oncology (Lisby et al. 2022). Its ability to resolve time-dependent drug effects, detect early cytostatic versus cytotoxic responses, and monitor migratory and invasive behavior in real time has made it an increasingly valuable tool in cancer biology and preclinical drug development (Evans et al. 2019; Hamidi et al. 2017). Moreover, the label-free nature of the RTCA system embraces high repeatability, consistency, and dynamic monitoring but eliminates the requirement for fluorescent or radioactive tags, thereby mitigating potential experimental artefacts and streamlining workflows for high-throughput screening and long-term kinetic studies (Kanemaru et al. 2022; Miceli et al. 2019; Yan et al. 2018a, b). The automated capabilities of the system significantly reduce the need for manual intervention, which not only enhances reproducibility but also facilitates the execution of large-scale experiments essential for pharmaceutical and biotechnological applications (Chiu et al. 2017). Despite this growing body of evidence, applications of RTCA in cancer research remain fragmented across experimental contexts, and a consolidated synthesis of how kinetic impedance signatures inform mechanistic and translational understanding is lacking. Hence, this review.

Beyond the xCELLigence framework, alternative commercial impedance systems and techniques have driven key advancements in cancer research. These include the ECIS^®^ platform (Applied BioPhysics) for cellular profiling (Moghtaderi et al. 2024), Electrical Impedance Spectroscopy (EIS) for monitoring chemoresistance (Crowell et al. 2020), the Maestro modules (Axion BioSystems) for oncology kinetics (Higgins and Gomillion 2025; Logun et al. 2023), and legacy systems such as the CellKey™ platform (MDS Analytical Technologies) (McGuinness 2007) and the RT-CES^®^ system (ACEA Biosciences). While these technologies have shaped the broader impedance-based landscape, we focus extensively on studies employing the principles of the xCELLigence platform.

## Principles of xCELLigence^™^ RTCA system

**Figure 1:**
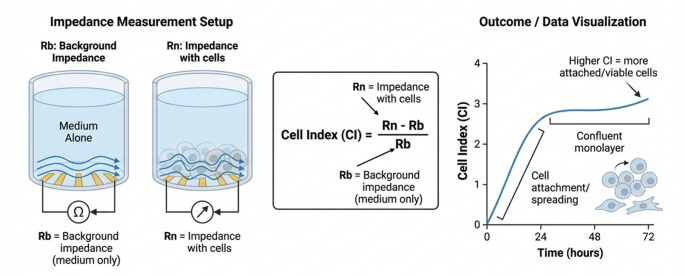
The impedance is converted into a dimensionless CI, calculated as CI = (Rn - Rb) / Rb, where Rn is the impedance with cells and Rb is the background impedance of the medium alone. Impedance-based real-time cell analysis quantifies dynamic changes in cell adhesion, morphology, and proliferation by monitoring the electrical impedance at microelectrodes integrated into the culture substrate. In this system, the raw impedance signal measured in wells containing only culture medium (background) is compared to the impedance in wells seeded with cells (Rn), and transformed into a dimensionless CI. As cells attach, spread, and form a confluent monolayer over the electrode surface, they partially insulate the current path and increase the impedance, resulting in a progressive rise in CI over time. Conversely, loss of adhesion, cytotoxicity, or cell death reduces electrode coverage and decreases CI. Thus, CI serves as an integrated biophysical readout of cell number, adhesion strength, and morphology, enabling label-free, kinetic assessment of cellular responses to stimuli such as LPS, drugs, or gene perturbations with high temporal resolution

The RTCA system comprises a computer with RTCA control software, an impedance analyser (depending on the model), specialized plates like the E-Plate or CIM-Plate with gold microelectrodes, and an RTCA station that holds these plates inside a tissue culture incubator at 37 °C and 5% CO_2_. Each well in these plates has gold biosensors covering about 80% of the bottom surface and creating an electric field when a low voltage is applied in the presence of cell culture medium or buffer. Adherent cells on the electrodes impede electron flow, increasing impedance proportional to cell attachment and spreading (Figure 1) (Hamidi et al. 2017; Khan et al. 2025). Real-time impedance measurements are converted into the CI, a dimensionless parameter that reflects changes in cell number, morphology, and adhesion (Khan et al. 2025). A CI of zero indicates no cell attachment, while higher values reflect increased cell coverage. The system continuously monitors CI, producing a kinetic profile or cellular fingerprint that captures dynamic cellular behaviours such as attachment, proliferation, detachment or death.

Standard E-plates feature gold biosensors embedded in glass-bottomed wells; however, polyethylene terephthalate (PET) plates are also available (Table 1). The E-Plate VIEW integrates biosensor-based monitoring with visual inspection, featuring a sensor-free window that correlates impedance signals with changes in cell number or size. The CIM-Plates, designed exclusively for the xCELLigence RTCA DP (dual purpose) system (Table 1), monitor cell invasion and migration in real time using electronically integrated Boyden chambers. As cells migrate from the upper chamber toward a chemoattractant in the lower chamber through 8 μm pores, they adhere to gold biosensors, generating a signal proportional to the number of attached cells, ensuring reproducible data collection. The E-Plate inserts facilitate co-culture studies, enabling real-time analysis of indirect cell-cell interactions while keeping cell types in separate compartments, thereby supporting diverse applications under physiological conditions.

The xCELLigence RTCA S16, the smallest model in the xCELLigence family, is compatible with E-Plate 16, E-Plate VIEW 16, and E-Plate 16 PET (Table 1). The RTCA Pro 2.8.1 software Lite is designed for RTCA S16 and provides predefined assay templates for easy experimental setup. Other xCELLigence models include: (1) the RTCA SP (single plate) system using 1 × 96-well format with rapid measurement of average read time of approximately 7 s; and (2) RTCA MP (multiple plates), which run up to six 96-well plates simultaneously or independently to maximise productivity (http://www.agilent.com, accessed September, 2025). The RTCA S16, SP and MP are used for analysing cell characterisation, immunotherapy/cell killing, viral effects, cytotoxicity, adhesion, barrier function, and signalling. On the other hand, the RTCA-DP system is designed to run up to three 16-well plates simultaneously or independently and is compatible with E-Plate 16, E-Plate VIEW 16, or CIM-plate 16. The system is used for measurement of cell number, size, morphology, and attachment properties, with the ability to perform kinetic analysis of cell invasion/migration (http://www.agilent.com, accessed September, 2025). Additional models include the RTCA HT (High Throughput) with 384-well format, which can accommodate up to four instruments and can be integrated and controlled by a single control unit; the HT-BioTek BioSpa8, which can accommodate up to eight 384-well E-Plates; the RTCA eSight, which combines impedance with bright-field and fluorescence imaging and which is currently the only instrument that provides both live cell imaging (label) and impedance (label-free) readouts of cells, both as adherent monolayer cells or as 3D tumour spheroids; the RTCA Cardio for monitoring cardiomyocyte beating in real-time for cardiotoxicity assessment and the RTCA Cardio ECR, designed for assessing cardiomyocyte contractility, viability, and electrophysiology (Khan et al. 2025).

**Table 1:** RTCA models with compatible plates and primary uses

RTCA Model	Plates	Primary Use Cases
S16	E-Plate (view) 16 (glass/PET)	Cytotoxicity assays, dose-response curves, IC_50_ calculation of small-scale studies
SP	E-Plate (view) 96 (glass/PET)	Cytotoxicity assays, dose-response curves, IC_50_ calculation of larger studies
MP	E-Plate (view) 96 (glass/PET) x 6	Cytotoxicity assays, dose-response curves, IC_50_ calculation of high-throughput studies
DP (Dual Purpose)	E-Plate (view) 16 (glass/PET) x 3; CIM-Plate 16 × 3	Cytotoxicity assays, dose-response curves, IC_50_ calculation; accommodates co-cultures; assessment of migration, and invasion.
HT	E-Plate 384 PET	Large-scale screening of compound libraries
HT-BioTek BioSpa8	E-Plate 384 PET	Large-scale screening of compound libraries using Agilent BioTek BioSpa 8 automated incubator. Maximum eight 384-well E-Plates
eSight	E-Plate 96, CIM-Plate 16	Correlating kinetics with cell morphology. Built-in inverted light and fluorescence microscope.
Cardio (ECR)	E-Plate CardioECR 48 or (view) 96	Cardiomyocyte viability, contractility, and electrophysiology (ECR model). Combines impedance with multi-electrode array (MEA) technology.

## Data interpretation: quantitative metrics and cellular fates

Beyond simple visualization of CI curves, the platform enables extraction of multiple quantitative metrics that provide time-resolved insights into cellular behaviours, functions, and fates including cytotoxicity, adhesion, proliferation, migration, and invasion. Assessment of these cellular fates, whether for effector or target cells or both, is critical in cancer research (Kouri et al. 2025).

### Proliferation, cytotoxicity, and dose-response

Assessing cell proliferation provides an immediate, real-time assessment of the toxicity of the cancer intervention being studied (Şener et al. 2017). In contrast to conventional endpoint assays, which report a single IC₅₀ or EC₅₀ value at a fixed time point, real-time cell analysis enables the determination of inhibitory or effective concentrations as a function of time (Phan et al. 2025). This temporal resolution allows the detection of delayed cytotoxic effects, transient cytostatic responses, and adaptive cellular recovery that are frequently overlooked by static measurements. As a result, time-dependent IC₅₀ and EC₅₀ profiling provides a more refined and biologically meaningful evaluation of therapeutic efficacy and the emergence of drug resistance (Sánchez-Díez et al. 2025).

### Growth kinetics and slope analysis

With impedance-based real-time cell analysis, evaluation of CI slope constitutes a core quantitative metric for interpreting growth kinetics and treatment response (Witzel et al. 2015; Khan et al. 2025). The slope of the CI curve $\left(\frac{{\Delta}\mathrm{C}\mathrm{I}}{{\Delta}\mathrm{t}}\right)$reflects the rate of impedance change over time and integrates dynamic alterations in cell proliferation, adhesion, morphology, and survival at the electrode interface. Steep positive slopes indicate rapid growth or migration, whereas flattened or negative slopes indicate growth inhibition or cell death. These kinetic parameters allow quantitative comparison of treatment effects across conditions (Lebourgeois et al. 2018). Unlike absolute CI values, which may be influenced by initial seeding density or baseline adhesion, slope analysis captures relative kinetic changes that more accurately represent biological behaviour under experimental perturbation. The Endpoint Migration Index and time to migration/invasion onset are also often used as quantitative endpoints to allow inter-study comparisons. Within co-culture scenarios in cancer immunology, the Effector: Target (E: T) ratio of cells is easily calculated based on the absolute number of cells seeded at the start of the co-culture period. Positive and sustained slopes are typically associated with active proliferation and stable cell attachment, whereas attenuated or negative slopes indicate growth inhibition, cytotoxicity, or progressive loss of adhesion (Su et al. 2025). Importantly, slope analysis enables temporal segmentation of cellular responses, allowing early, intermediate, and late phases of treatment effects to be distinguished. For example, an initial reduction in slope followed by partial or complete recovery may reflect transient cytostatic stress or adaptive resistance, while a persistent decline is more consistent with irreversible cytotoxicity. These distinctions are often obscured in endpoint assays that collapse complex temporal responses into a single measurement.

When interpreted alongside time-dependent concentration–response relationships, CI slope analysis enhances mechanistic analysis into therapeutic action by linking kinetic behaviour to dose, exposure duration, and cellular adaptation (Hoare et al. 2021; Nguyen et al. 2023). As such, slope-based growth kinetics represent a robust and informative component of impedance-derived quantitative analysis, strengthening both biological interpretation and translational relevance in cancer research.

### Migration, invasion, and metastatic potential

Understanding cell invasion and migration is critical for elucidating the molecular and biological mechanisms underlying cancer metastasis. This involves changes in cell shape and behaviour through epithelial-mesenchymal transition (EMT). Cell invasion involves the penetration and degradation of adjacent tissues, often associated with cancer cells, while cell migration refers to the directed movement of cells, typically in response to chemical signals, playing a key role in processes like cancer metastasis and tumour progression (Li et al. 2025). The use of CIM-Plates in real-time cell analysis enables continuous, label-free monitoring of cancer cell migration and invasion, eliminating the need for endpoint staining or manual cell quantification (Bird and Kirstein 2009; Martínez-Serra et al. 2014). This approach captures the full temporal profile of motility, allowing precise determination of migration onset, rate, and extent. Parameters such as onset time and cell index slope provide insight into early signalling events and cytoskeletal reorganization, while the endpoint migration index reflects cumulative invasive capacity. Together, these complementary metrics improve reproducibility and support more mechanistic interpretation of motility-related phenotypes, particularly in contexts where therapeutic interventions exert time-dependent or transient effects on cell movement.

### Adhesion, barrier integrity, and EMT

Assessment of cancer cell adhesion is also fundamental in modulating important processes like cell–cell communication, cell proliferation, survival, migration, and differentiation (Hamidi et al. 2017). Adhesion assays capture the kinetics of early integrin-mediated attachment. Furthermore, barrier integrity assays quantify the formation and disruption of tight epithelial monolayers, where a decrease in CI is directly proportional to the loss of cell-cell barrier functions. The system also tracks EMT-related morphological transitions, such as the loss of epithelial adhesion and acquisition of mesenchymal motility, as characteristic shifts in impedance signatures.

### Effector–target dynamics in immunotherapy assays

In co-culture systems, real-time cell analysis enables continuous assessment of effector cell-mediated cytotoxicity against adherent tumour targets. Changes in cell index over time reflect the balance between tumour cell survival, detachment, and death following immune cell engagement (de Visser and Joyce 2023). By correlating impedance kinetics with defined effector-to-target ratios, this platform permits quantitative evaluation of immune killing dynamics, including onset of cytotoxic activity, rate of target cell elimination, and durability of response. Such kinetic resolution provides insight into functional immune potency and tumour susceptibility that cannot be captured by single-time-point cytotoxicity assays, thereby strengthening the translational relevance of in vitro immunotherapy evaluation (Sanjai et al. 2024).

**Figure 2:**
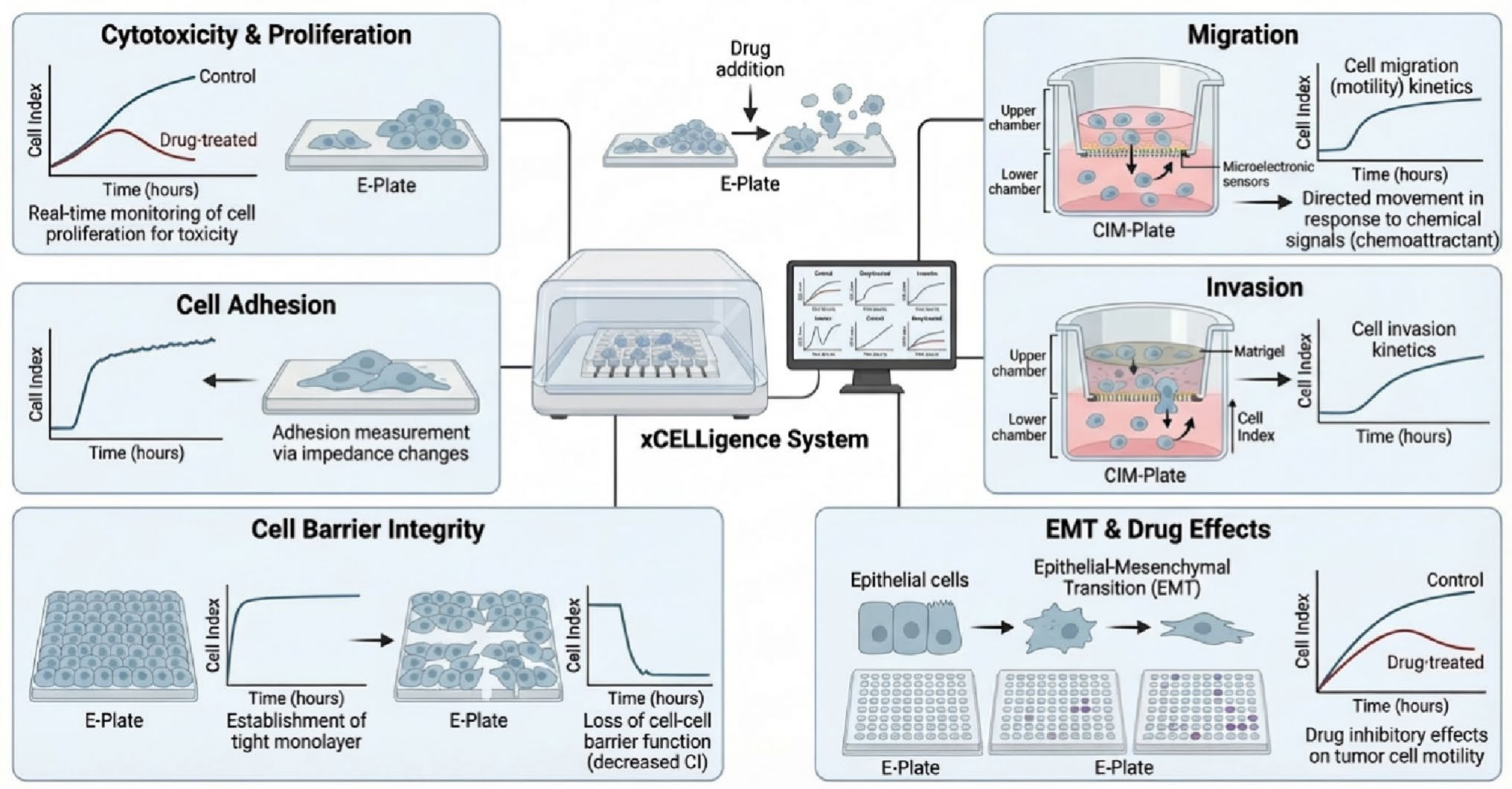
xCELLigence System: multiple cellular functions assessed in cancer research. Various cancer therapeutic interventions resulting in different cellular fates. This illustration broadens the scope of impedance‑based real‑time cell analysis by showing how a single biophysical readout can be deconvoluted into multiple functional phenotypes relevant to cancer biology. Because impedance reflects the degree to which cells adhere, spread, migrate, or form barrier structures on microelectrode‑embedded plates, the xCELLigence system can continuously quantify diverse behaviors without labels or endpoint staining. In proliferation and cytotoxicity assays, rising or falling Cell Index values track drug‑induced growth inhibition or cell death with minute‑scale resolution. Adhesion assays capture the kinetics of integrin‑mediated attachment, revealing how compounds modulate early anchorage events. Migration and invasion assays use CIM plates with porous membranes to measure chemotactic movement or matrix penetration, translating impedance changes into motility rates. Barrier integrity assays quantify the formation and disruption of tight epithelial monolayers, enabling detection of permeability changes associated with inflammation, EMT, or drug toxicity. Finally, EMT‑related morphological transitions such as loss of epithelial adhesion and acquisition of mesenchymal motility as characteristic shifts in impedance signatures. Together, these modalities demonstrate how real‑time impedance profiling provides a unified, high‑resolution platform for dissecting therapeutic responses, cellular plasticity, and microenvironment‑driven behaviors in cancer research

The following sections will summarise recent advances in cancer research where different therapeutic interventions (ranging from gene/pathway inhibitions to immunotherapy and nanotherapy) have been utilised, resulting in various cellular fates (e.g., proliferation, invasion/migration, etc.) assessed by the xCELLigence system (Figure 2).

## Cancer therapy interventions assessed using xCELLigence RTCA

The sections below address cancer therapy interventions in which the xCELLigence system was invaluable in assessing various cellular behaviours and fates.

### Kinase inhibition

Kinase inhibition has become a cornerstone of modern cancer therapy, representing a significant shift from traditional chemotherapy toward targeted, more precise treatments. Kinases are involved in processes such as cancer cell proliferation and survival, angiogenesis, as well as metastasis, migration, and invasion. The xCELLigence has proven valuable in assessing the effectiveness of kinase inhibitors in either resulting in cancer cell death or a reduction in migration. For example, Uzunhisarcıklı (2024) evaluated the cytotoxic and antiproliferative effects of bronchodilators and kinase inhibitors, terbutaline and orciprenaline, on A549 lung cancer cells (12,500 cells per well) and normal BEAS-2B cells (10,000 cells per well) using the xCELLigence RTCA system. Terbutaline and orciprenaline inhibit Glycogen synthase kinase 3β (GSK3β), which contributes to the pathogenesis and progression of several diseases, including cancer (Domoto et al. 2020). GSK3 inhibitors are known to interfere with abnormal activation of signaling pathways in cancer cells, leading to cell cycle arrest, inhibition of proliferation and induction of apoptosis (Thapa et al. 2023). Cells were treated with 1 to 400 µM of pemetrexed (positive control), terbutaline, and orciprenaline, and monitored for 72 h. Both agents inhibited A549 cell proliferation, with orciprenaline showing greater efficacy, while exhibiting low cytotoxicity on normal BEAS-2B cells, thereby suggesting selective anticancer potential. On the other hand, the xCELLigence RTCA DP system was used to assess the inhibition of migration and metastatic processes of cystic fibrosis pancreatic ductal adenocarcinoma (CFPAC-1) cells through the use of danusertib, a third-generation Bcr-Abl tyrosine kinase and pan-Aurora kinase inhibitor (Alabaş and Özçimen 2024). Using CIM-16 plates with a cell density of 20,000 per well, the researchers demonstrated that danusertib significantly suppressed the in vitro migration of these highly aggressive cancer cells.

### Gene silencing

Gene silencing offers a precise and targeted way to interfere with the specific molecular mechanisms that drive cancer. Unlike traditional chemotherapy, gene silencing aims to selectively shut down the genes that are essential for the survival or migration of cancer cells or their resistance to treatment. The use of the xCELLigence RTCA in assessing the efficacy of gene silencing in the inhibition of cancer migration is indeed prevalent based on recent literature. For example, Escalona et al. (2022) utilised the RTCA system to investigate the role of endogenous tissue inhibitor of metalloproteinase-2 (TIMP-2) in OVCAR5 ovarian cancer cell migration. TIMP-2 in cancer cells, through its homeostatic action on certain metalloproteinases, plays a vital role in remodelling the extracellular matrix (ECM) to facilitate cancer progression. The researchers reduced TIMP-2 expression via siRNA or CRISPR/Cas9, generating transient and stable knockdowns with single or pooled TIMP-2 siRNAs (T2-KD cells) or CRISPR/Cas9 with two distinct guide RNAs (gRNA1 and gRNA2). Migration and invasion assays using the xCELLigence RTCA demonstrated altered motility in TIMP-2-deficient cells (at 40,000 cells per well), highlighting TIMP-2 as a potential therapeutic target in gene silencing approaches. In another study, the xCELLigence was used to identify the role of TAp73 in suppressing the metastatic potential of PDAC (Ungefroren et al. 2023). In a chemokinesis setup, PANC-1 cells were transiently transfected twice with 50 nM control siRNA (siCo) or TAp73 siRNA (siTAp73) and monitored for migration with or without exogenous TGF-β1 (5 ng/mL). Impedance measurements revealed enhanced migration upon TAp73 silencing, particularly in the presence of TGF-β1.

In another gene silencing study, Phatak et al. (2022) used the xCELLigence RTCA to identify RAB14 expression as a potential therapeutic target in oesophageal cancer. Oesophageal cancer cell lines (hESO, TE7, FLO-1, and SK-GT-4) (40,000–80,000 per well) were transfected with anti- or pre-miR-214-3p (tumour suppressor) or RAB14 (oncogenic membrane trafficking protein) plasmids for 48 h, and migration and invasion were assessed. Forced expression of miR-214-3p or RAB14 silencing significantly reduced cancer cell migration and invasion.

#### Disruption of the glycolytic pathway

Targeting the glycolytic pathway, a key metabolic vulnerability of many cancer cells, represents a promising and therapeutically relevant strategy in oncology. The Sodium-Glucose Linked Transporter 2 (SGLT2) protein is a glucose transporter often overexpressed in cancer cells, allowing increased glucose uptake referred to as the “Warburg effect” (Basak et al. 2023). Inhibition of SGLT2 has shown promise in cancer therapy. Using the xCELLigence RTCA, Karzoon et al. (2025) investigated the anticancer effects of empagliflozin and tamoxifen, both SGLT2 inhibitors on MCF-7 breast cancer cells. Cells seeded at 15,000 per well in E-Plates were treated with empagliflozin (IC_50_ = 177 µM) and tamoxifen (IC_50_ = 17 µM), individually or combined. Time- and concentration-dependent cytotoxic effects were observed, with synergistic effects at concentrations near their IC_50_ values, suggesting enhanced therapeutic potential for ERα + breast cancer treatment. Similarly, Pawlos et al. (2023) investigated the effects of SGLT2 inhibitors, empagliflozin, canagliflozin, and dapagliflozin. The xCELLigence RTCA was not used for cytotoxicity assessment, but rather for human umbilical vein endothelial cell (HUVEC) barrier integrity as the endothelial barrier integrity plays a crucial role in regulating vascular permeability, which in turn can influence tumour growth and metastasis The study found that all three SGLT2 inhibitors significantly improved the integrity of the endothelial cells, which had been compromised by 25-hydroxycholesterol (25-OHC), a proatherogenic stimulus. At the 24-hour mark, canagliflozin and dapagliflozin showed a particularly strong effect, improving integrity by 97.8% and 111%, respectively, compared to the 25-OHC control.

Another study showed the potential of the xCELLigence RTCA to assess cancer cell substrate attachment by inhibiting the glycolytic pathway (Fan 2024). Cell-substrate attachment refers to the ability of cells to adhere to the surface they are cultured on, which is crucial for maintaining structural integrity, proliferation, and overall cell function. The study revealed that at low concentrations of 3-(3-pyridinyl)-1-(4-pyridinyl)prop-2-en-1-one (3PO), an inhibitor of the glycolytic enzyme, 6-Phosphofructo-2-Kinase/Fructose-2,6-Biphosphatase 3 (PFKFB3), colorectal cancer (CRC) cells maintained their cell-matrix adhesion quality. However, as the concentration of 3PO increased to 25 µM, 50 µM, and 75 µM, there was a notable decline in the quality of cell-substrate attachment, which in turn resulted in a significant reduction in cell proliferation. The alterations in cell-substrate attachment in response to 3PO suggest that the inhibitor not only affects cell proliferation but may also disrupt the fundamental processes that allow cancer cells to adhere and thrive in their microenvironment, as was evidenced by the xCELLigence RTCA. This disruption could be leveraged in therapeutic strategies targeting CRC.

The research demonstrates that targeting a specific pathway provides a multifaceted approach for evaluating diverse cancer cell responses to therapeutic interventions, including cytotoxicity, vascular integrity, and cell substrate attachment.

#### Targeting the intrinsic mitochondrial pathway

Although the intrinsic mitochondrial pathway is often assessed as confirmation of apoptosis as a downstream consequence, direct targeting of this pathway in cancer cells is also often a deliberate strategy in inducing anticancer effects. Raphela-Choma et al. (2024) examined the anticancer activity of iso-mukaadial acetate (IMA), a drimane sesquiterpenoid from *Warburgia salutaris* bark, on pancreatic (MIA-PACA2) and colon (HT29) cancer cells, as well as normal HEK293 cells, using the xCELLigence RTCA system. At 100,000 cells per well in E-Plates, IMA exhibited concentration- and cell-type-dependent cytotoxicity, with pronounced toxicity toward cancer cells at lower concentrations and broader toxicity at higher doses, supporting the potential of IMA as a selective anticancer agent. The anticancer activity is due to alteration of the mitochondrial intrinsic route in cancer cells involving MMP disruption, caspase 3/7 activation, cellular ATP decrease and Bax/Bcl2 expression. Similarly, Rajabathar et al. (2023) evaluated the antiproliferative effects of herbal melanin on human breast adenocarcinoma (MDA-MB-231) and colorectal (HCT 116) cells using the xCELLigence RTCA system. Cells seeded in E-Plates (MDA-MB-231, 5,000 cells/well and HCT 116, 12,000 cells/well) were treated with various concentrations of herbal melanin. Impedance measurements revealed significant inhibition of cell proliferation, suggesting the potential of herbal melanin as an alternative anticancer agent for these cancer types. The anticancer activity involved downregulation of Bcl-2 and Bcl-xl, upregulation of p53 expression, downregulation of Bcl-2, Bcl-xl, XIAP and Survivin, upregulation of Bid, Bax, p53, Cytochrome C and PARP apoptotic protein expression and lastly, upregulation of caspase 3 and 9 expressions.

#### Epithelial-to-mesenchymal transition (EMT) targeting

Targeting EMT is a crucial strategy in cancer research as it directly interferes with the processes that drive metastasis, drug resistance, and tumour recurrence. Basmaeil et al. (2024) evaluated the anticancer effects of decidua basalis mesenchymal stem cells (DBMSCs) on MDA-MB-231 and MCF-7 breast cancer cell lines by decreasing EMT-related protein levels in MDA231 cells and modulating expression of other cancer-related genes in MDA231 and MCF7 cells. Using xCELLigence CIM plates to monitor migration (20,000 cells per well, with 10% foetal bovine serum as a chemoattractant, recorded over 24 h) and E-Plates to assess invasion (10,000 cancer cells seeded onto HUVEC monolayers, monitored for 48 h), the study showed that DBMSCs inhibited both migration and invasion. Additionally, proliferation and adhesion were evaluated over 72 h using E-Plate 16, with the first two hours reflecting adhesion and the full duration capturing proliferation dynamics. These findings underscore the ability of the xCELLigence to comprehensively assess multiple cellular parameters, providing a robust platform for studying complex anticancer mechanisms. Using the xCELLigence RTCA, Bai et al. (2025) demonstrated the concentration-dependent inhibition of 4T1 breast cancer cell proliferation (seeded at 5,000 cells/well in E-Plates) by *Rhodiola rosea *L., a traditional Chinese medicinal herb. Its anti-tumour effects involved downregulating the expression of HIF-1α, TGF-β, and Smad3 in tumour cells, thereby inhibiting EMT and reducing immunosuppression of immune cells. The RTCA assays confirm that targeting key signalling pathways to inhibit EMT effectively prevents the cellular plasticity required for cancer cells, thereby resulting in a decreased risk of metastasis.

#### Combining natural compounds with traditional chemotherapy

Combining natural compounds with traditional chemotherapy is a good strategy for cancer therapy for several reasons, primarily due to the potential for synergy, the ability to reduce side effects, and the capacity to overcome drug resistance. For example, Yılmaz and Özdemir (2024) used the xCELLigence RTCA system to investigate the cytotoxic effects of oleuropein, a natural polyphenol from olives, combined with paclitaxel on MCF-7 breast cancer cells. Cells seeded at 8,000/well in E-Plates were treated with oleuropein and paclitaxel at various ratios (100%, 75%, 50%, 40%, 30%, 20%, 10%) based on their IC_50_ doses. High-dose combinations (100%, 75%, 50%) showed antagonistic effects, while low-dose combinations (40%, 30%, 20%, 10%) exhibited synergistic cytotoxicity, reducing the effective paclitaxel dose below its IC_50_ (7.5 µM) and highlighting the potential to enhance chemotherapy efficacy while minimising toxicity. Although the precise mechanism in which oleuropein exerted its antitumor effects is unknown, it was postulated that it led to an increased antioxidant defence and decreased Oxidative Stress Index levels. Nevertheless, the quantitative assessment by the RTCA system was essential for identifying the precise concentration ratios that transitioned from antagonistic to synergistic effects. In another study, Kul Köprülü et al. (2025) evaluated the cytotoxicity of royal jelly (RJ) combined with paclitaxel on oral squamous carcinoma cells (UPCI-SCC-131) and healthy gingival HGF cells using the xCELLigence RTCA system. Impedance measurements demonstrated that both RJ and paclitaxel exerted significant antiproliferative effects on cancer cells, while RJ co-treatment reduced paclitaxel-induced cytotoxicity in healthy cells, suggesting a protective role against chemotherapy-associated toxicity and enhancing the anticancer efficacy of paclitaxel.

### Immunotherapy

xCELLigence RTCA systems are also a powerful tool for evaluating immunomodulatory responses. This technology enables the continuous monitoring of immune cell-mediated killing and cytotoxicity without the need for labels, which is particularly useful for developing immunotherapies and assessing immune cell function (Yan et al. 2018a, b). The studies addressed below demonstrate the use of RTCA in evaluating immune cell activity in cancer research.

#### CAR-T cells

Chimeric antigen receptor (CAR)-T cells offer a significant advantage over conventional chemotherapy by providing a highly targeted therapeutic approach. While traditional chemotherapy can indiscriminately damage both cancerous and healthy cells, CAR-T cells are engineered to specifically recognise and eliminate malignant cells. This precise targeting enhances the efficacy of the treatment and minimises off-target toxicity, making it a particularly potent strategy for managing specific hematological malignancies. For example, Valiullina et al. (2023) investigated the cytotoxic effects of CAR-T cells against MCF-7 breast cancer cells and modified MCF-7(CD19+) cells using the xCELLigence RTCA system. MCF-7 and MCF-7(CD19+) cells, seeded at 5,000 cells per well in E-Plates, were treated with CAR-T cells or control T cells, and proliferation and cytotoxicity were monitored in real time. The study demonstrated significant CAR-T cell-mediated cytotoxicity against the modified MCF-7(CD19+) cells, underscoring the potential of adoptive T-cell therapy for breast cancer treatment.

#### NK cells and ADCC

The targeting of tumour surface antigens with monoclonal antibodies (mAbs) is a cornerstone of modern cancer therapy. One key mechanism is antibody-dependent cell-mediated cytotoxicity (ADCC), where antibodies activate immune effector cells, such as natural killer (NK) cells, to kill cancer cells. A study by Jiao et al. (2021) used the RTCA system to investigate the cytotoxic effects of NK cells on A549 lung cancer cells. They found that both normal and senescent A549 cells were killed by NK cells, but there was no significant difference in cytotoxicity between the two cell types at the same effector-to-target (E: T) ratio. However, the study also explored the role of an anti-LUNX antibody in enhancing NK cell-mediated killing of senescent A549 cells. RTCA data showed that senescent A549 cells co-cultured with NK cells and the anti-LUNX antibody were killed more efficiently than those without the antibody after 24 h. Normal A549 cells, on the other hand, didn’t show a significant difference until 36 h. This suggests that senescent A549 cells may be more responsive to the anti-LUNX antibody, likely due to increased LUNX expression on their surface. These findings indicate that inducing immunogenic senescence with FDA-approved drugs, combined with an anti-LUNX antibody, could be a new strategy for treating non-small cell lung cancer (NSCLC). Similarly, Cheng et al. (2021) used the xCELLigence RTCA system to demonstrate the strong, dose-dependent cytotoxicity exhibited by oNK-1 cells, an engineered NK cell line, against most cancer cell lines including ovarian, breast and lung cancers. Importantly, oNK-1 cells showed greater cytotoxicity against OVCAR-3 cells than primary donor-derived NK cells. The study also confirmed the ADCC activity of oNK-1 cells against ovarian cancer cells in the presence of trastuzumab, a commonly used antibody for HER2-positive cancers. The RTCA data revealed that oNK-1 cells had a higher ADCC activity than a standard NK-92 cell line, showcasing their potential as a therapeutic agent. Similarly, Li et al. (2021) used the xCELLigence system to assess the enhanced cytotoxicity of ACE-oNK-HER2 cells (oNK cells engineered with a proprietary technology) against HER2-positive cancer cells. They found that these engineered cells showed significantly higher cytotoxicity against SK-OV-3, SK-BR-3, and MCF-7 cancer cells compared to control oNK cells. The specificity of ACE-oNK-HER2 was confirmed by a lack of cytotoxicity against HER2-negative K562 cells and healthy donor PBMCs. The study also compared the ADCC activity of ACE-oNK-HER2 with control cells in the presence of trastuzumab, concluding that the engineering process provided superior cytotoxicity to ADCC alone. These findings highlight the potential for using engineered NK cells as “off-the-shelf” therapies for cancer.

Finally, the xCELLigence RTCA system can be used to study how immune cell-based therapies can overcome drug resistance. Baysal et al. (2020) used the xCELLigence system to analyse cetuximab-induced ADCC in head and neck squamous cell carcinoma (HNSCC) cell lines, including a cetuximab-resistant line. By co-culturing these cells with NK92 cells that either expressed (CD16+) or lacked (CD16-) the cetuximab-binding receptor, they confirmed that the increased killing effect was directly due to the interaction between cetuximab and CD16, thus confirming ADCC as the mechanism. These results demonstrated that cetuximab treatment combined with CD16 + NK cells could effectively overcome cetuximab resistance in one of the cell lines, highlighting the potential for combining cetuximab with immunotherapeutic approaches to enhance anti-tumour responses.

#### Bispecific T cell engagers (BiTE)

Bispecific T-cell Engagers (BiTEs) constitute a novel class of immunotherapeutic agents engineered to leverage the endogenous immune system for the targeted elimination of malignant cells. As a form of bispecific antibody, these molecules possess the unique capacity to bind simultaneously to two distinct cell surface antigens, thereby facilitating a cytotoxic interaction. Engineering BiTE immune cells has been shown new promise in cancer research. However, BiTE have several drawbacks i.e. they could lead to graft-versus-host disease (GvHD) and show rapid clearance from the circulation. Li et al. (2022) engineered T cells with a BiTE consisting of CD3ε scFv and a target-binding scFv tandemly linked together. These BiTE-engineered T (BiTE-T) cells show reduced reaction to T cell receptor stimulation in vitro and have low risk of GvHD in vivo. The xCELLigence RTCA was used to assess the efficacy of the BiTE T cells to kill target cancer cells, CD19 and Her2. In another study, Mandrup et al. (2021) could show via E: T challenge assays the ease of use of the xCELLigence system in assessing the efficacy of BiTE fused with albumin to increase its half-life within the circulation. In both these studies, the xCELLigence RTCA has proved valuable in assessing a novel form of cancer therapy.

#### Targeting tumour extracellular matrix (ECM) proteins

Cancer immunotherapy has identified the importance of cancer ECM proteins in influencing T cell dynamics, thereby suggesting that targeting these interactions could represent a novel therapeutic strategy to improve cancer treatment. Søgaard (2001) utilised xCELLigence RTCA to investigate the immunomodulatory effects of specific ECM proteins on T cell cytotoxicity against tumour cells. The study evaluated how different ECM proteins, particularly those overexpressed in tumour-associated fibroblasts, influenced the cytotoxic efficacy of autologous tumour-infiltrating lymphocytes (TILs) against patient-derived melanoma cells. The xCELLigence findings revealed that while many of the screened ECM proteins did not significantly alter TIL cytotoxicity or tumour cell proliferation, periostin (POSTN) notably enhanced TIL cytotoxicity by up to 80% when incubated with TILs both before and during co-culture with tumour cells. This suggests that POSTN may play a critical role in modulating the immune response within the tumour microenvironment, potentially enhancing the effectiveness of immunotherapy.

#### Oncolytic viruses

Oncolytic adenoviruses are highly promising vectors for oncolytic virotherapy (Farrera-Sal et al. 2021), noted for being easily genetically manipulated, stable, and historically safe as gene therapy vectors. These viruses are designed to selectively replicate within cancer cells and killing them, inducing inflammation, and promoting an immune response (epitope spread). Critically, they can also be engineered to act as targeted cancer gene therapy by encoding therapeutic transgenes like cytokines. This dual-action approach delivers high concentrations of therapeutic molecules directly to the tumour, thereby bypassing systemic toxicity (Kaufman et al. 2015). Again, the xCELLigence system proved useful in assessing oncolytic viruses in cancer therapy. For example, Ohnesorge et al. (2022) used T-VEC to treat NUT carcinoma. T-VEC is an oncolytic herpes simplex virus type 1 (HSV-1) talimogene laherparepvec, already approved for the treatment of melanoma. T-VEC harbours two copies of the human granulocyte-macrophage colony-stimulating factor (GM-CSF) gene, inserted into the ICP34.5/ICP47-deleted version of strain JS1, which may further enhance the virus-induced systemic anti-tumour immune response. Using the xCELLigence system, it was shown that T-VEC alone could successfully induce cytotoxicity in NUT cells. Park et al. (2020) engineered an oncolytic virus to express a non-signalling, truncated CD19 (CD19t) protein for tumour-selective delivery, enabling targeting by CD19-CAR T cells. Infecting tumour cells with an oncolytic vaccinia virus coding for CD19t (OV19t) produced *de novo* CD19 at the cell surface prior to virus-mediated tumour lysis. Using the xCELLigence system co-cultured CD19-CAR T cells secreted cytokines and exhibited potent cytolytic activity against infected tumour cells (human triple-negative breast cancer and glioblastoma), seeded at 20 000 cells/well.

#### Checkpoint inhibitors

Cancer therapy via checkpoint inhibition is a very effective form of therapy. Immune checkpoint inhibitors (ICI) targeting the PD-1/PD-L1 axis have paved the way in cancer treatment and the xCELLigence system has proved useful in this domain. For instance, Yang et al. (2020) could observe the real-time cytotoxic effect of CAR T cells engineered to target the programmed death-ligand 1 (PD-L1) expressed on PDAC cells. The addition of these genetically modified T cells at E: T ratios of 10 and 20 of PD1ACR-T or PDL1CAR-T cells alone resulted in an abrupt decrease in impedance, and CI values were significantly lower in PD-L1-high CFPAC1 cells compared with PD-L1-low Capan1 control cells. These results indicated that modified PD1ACR-T or PDL1CAR-T cells treated alone retained significant cytotoxic activities toward PD-L1-positive pancreatic tumours specifically.

Targeting only one checkpoint may often lead to cancer treatment resistance and combinatorial approaches are often suggested. For example, Muik et al. (2022) showed an improved model in which a bispecific antibody directed to both the PD-L1 checkpoint and 4-1BB showed enhanced anti-tumour activity (mbsAb-PD-L1 × 4-1BB). Binding of 4-1BB monoclonal antibodies to its natural ligand 4-1BBL induces clustering of 4-1BB and intracellular signalling, resulting in cellular activation, proliferation and cytokine secretion. 4-1BB represents an attractive immunotherapy target, as its engagement activates T-cell and NK-cell-mediated antitumor immunity. Using xCELLigence RTCA, the capacity of mbsAb-PD-L1 × 4-1BB to enhance T-cell mediated cytotoxicity was demonstrated on co-cultures of ovalbumin (OVA257-264) peptide-stimulated CD8 + OT-I T cells and ovalbumin-expressing B16 (B16_OVA-MO4) tumour cells. mbsAb-PD-L1 × 4 -1BB-induced T-cell-mediated cytotoxicity was similar to mAb- PD-L1 or the combination mbsAb-PD-L1×ctrl and mbsAb-ctrl×4-1BB, suggesting that cytotoxicity was enhanced predominantly by PD-L1 checkpoint blockade activity under these experimental conditions.

#### Combination therapies

Combination therapies are a cornerstone of modern cancer treatment, as they typically elicit a more potent and enduring response compared to single-agent regimens with regard to overcoming drug resistance, achieving synergistic effects and/or increasing efficacy while reducing toxicity. This review has previously touched on the use of the xCELLigence RTCA in assessing combinations of natural compounds with traditional chemotherapy. The xCELLigence RTCA has also proved valuable in combinations of different immune therapies. For example, Pakola et al. (2025) used a combination of oncolytic viruses, PD-L1, checkpoint inhibition and chemotherapy to decrease resistance to PDAC. The oncolytic virus, armed with normal human IL-2 (Ad5/3‑E2F‑d24‑vIL2) had a higher preferential binding to interleukin-2 receptor subunit beta, thus targeting the transgene to resting NK cells and naïve T cells and therefore avoiding expansion of T regulatory cells. This would allow direction of the early immune response away from an immunosuppressive microenvironment present in many tumours such as PDAC. In addition, anti-PD-L1 atezolizumab was used for checkpoint inhibition as well as chemotherapy in the form of nanoparticle albumin-bound paclitaxel (nab-paclitaxel) and gemcitabine. They used the xCELLigence system to test the combination therapy ex vivo, i.e. on human PDAC samples collected fresh from surgery (HUSPDAC-01 to HUSPDAC-03) and seeded at 50,000 cells/well. All three samples showed significantly enhanced killing in groups where Ad5/3-E2Fd24-vIL2 was present. Depending on the sample, anti-PD-L1 alone had minimal effect on cell killing, mimicking the effects seen in the clinic. This shows the use of the xCELLigence in assessing combination therapy ex vivo.

Conte et al. (2025) used an in vitro combination of IL-13Rα2 targeted CAR T-cells with an oncolytic virus (herpes simplex virus (HSV)-based oncolytic virus C134) in order to improve CAR T-cell efficacy towards PBT030 neurospheres (human glioblastoma). The cells were seeded at 20 000 cells per well and the xCELLigence system was used to assess the efficacy of the combined approach in terms of its toxicity on cancer cells.

### Nanomedicine and emerging therapeutic modalities

Nanomedicine offers a transformative approach to cancer therapy by utilising nanomaterials (NMs) to overcome the limitations of conventional treatments (Bor et al. 2019). A primary advantage is enhanced targeted drug delivery, where NMs can be engineered to accumulate at tumour sites, either passively through the Enhanced Permeability and Retention (EPR) effect or actively by binding to cancer-specific receptors. This precision minimises systemic toxicity and damage to healthy cells, thereby reducing severe side effects. Furthermore, nanomedicine can improve pharmacokinetics and bioavailability by protecting drugs from premature degradation, increasing their solubility, and extending their circulation time, which allows for more effective drug concentrations at the tumour (Malik et al. 2023).

Another key benefit is the ability to develop multifunctional therapeutic and diagnostic platforms, often referred to as theranostics. These integrated systems can combine diagnostic imaging agents with therapeutic components on a single NM, enabling real-time monitoring of treatment efficacy and facilitating highly personalised medicine. Lastly, nanomedicine provides a powerful tool for overcoming drug resistance. By co-delivering multiple agents with different mechanisms of action or by enabling the delivery of agents that can bypass resistance pathways, nanomedicine can lead to more durable and effective responses.

The xCELLigence technology is also suitable for nanotherapy applications due to its reliance on a label-free detection method. This is particularly advantageous because conventional label- and colour-based assays, such as the widely used MTS or MTT assays, are prone to interference from NMs. NMs may directly interact with the dyes and reagents used in these assays, leading to inaccurate and confounding results, including false positives or negatives (Andraos et al. 2020). In contrast, since the xCELLigence technology does not require the addition of external dyes or labels, it circumvents the inherent challenges posed by NM interference, enabling a more reliable and precise assessment of nanotherapy efficacy and cytotoxicity.

In the study by Géloën et al. (2021), the xCELLigence system was used to assess the cytotoxic effect of fluorescent carbon fluoroxide (CFO) nanoparticles (NPs) on cancerous and non-cancerous cell lines before and after washout of the NPs from the cell culture medium. CFO NPs have potential uses in bioimaging and cancer theranostic applications. The 3T3-L1 (non-cancer), HuH7 (human hepatocellular carcinoma), Panc1 (pancreatic cancer), HSC-2 (human oral squamous carcinoma), HepG2 (hepatocellular carcinoma) and HEK 293 (non-cancer) cell lines were seeded at 2500 per well in a 96 well E-plate. Their results show more toxic effects of the CFO NPs on cancer than on non-cancer cell lines. Due to the successful implementation of the xCELLigence the authors could conclude that cytotoxicity effects of NPs should be studied not only during their direct exposure to cells but also after their washout from the culture medium. In another study by Garcia et al. (2023), the xCELLigence was used to assess the anti-tumoral effects of silver NPs on mouse CT26 colon carcinoma and MCA205 fibrosarcoma cells. The RTCA system also showed promising cytotoxicity results of gallium- and indium-based NPs toward human bronchial epithelial cells (Nguyen et al. 2020). The effectiveness of NMs as carriers of anticancer agents has also been evaluated using the system. For example, using xCELLigence, Jafari et al. (2022) assessed the cytotoxicity and gene expression effects of curcumin-piperine loaded NPs on the breast cancer cell line MDA-MB-231 and compared it to that of docetaxel. The IC_50_ values of the NPs were higher than the IC_50_ value of docetaxel. Similarly, Koc-Bilican et al. (2025) used the RTCA system to show a dose and time-dependent suppression of cell proliferation of breast cancer cells by a Thymus vulgaris-loaded giant macroporous silica (GMS)-gold NP system.

**Figure 3:**
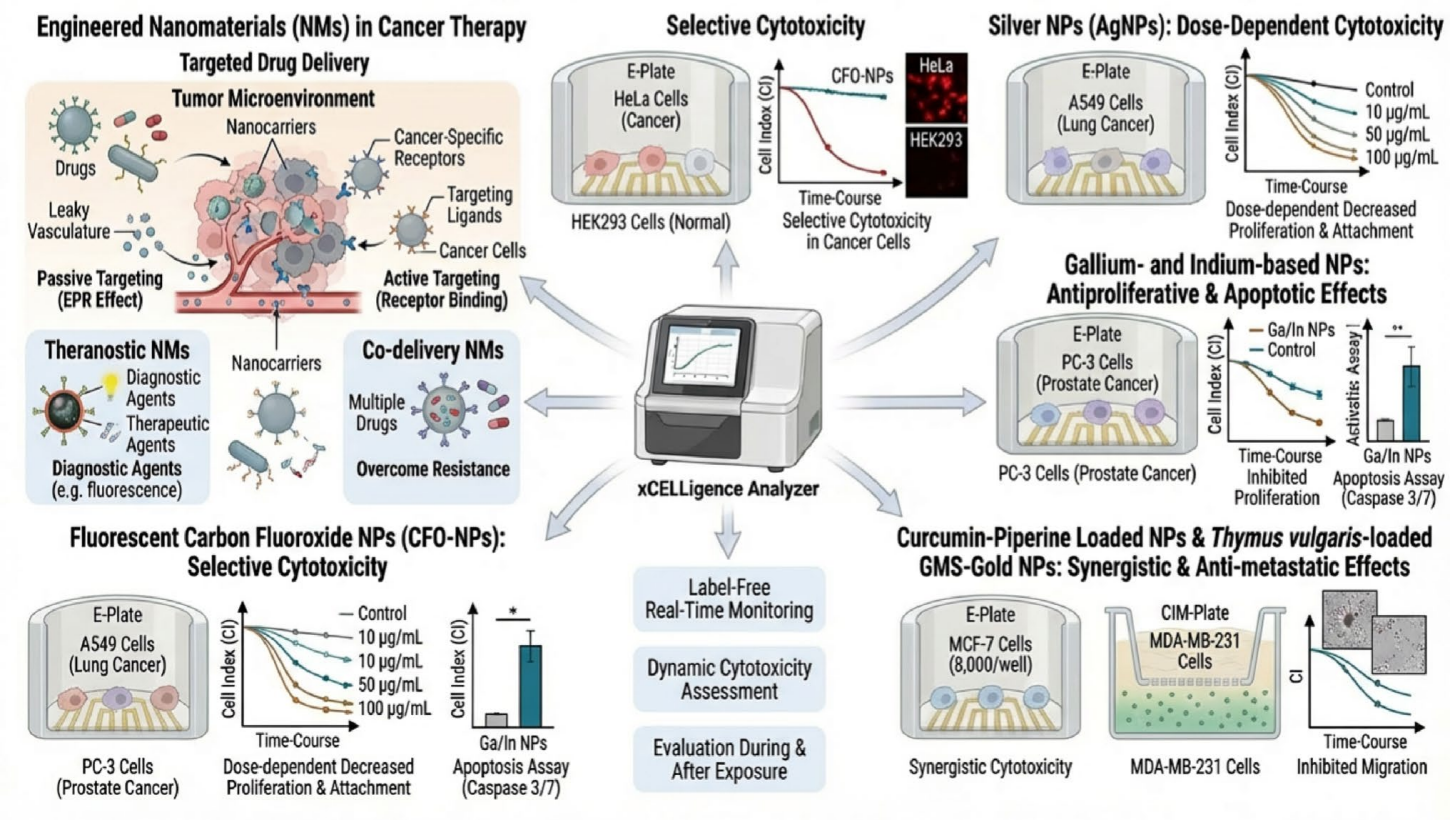
RTCA, Engineered Nanomaterials, and Cancer Therapy. The xCELLigence system enables continuous, label-free monitoring of cell proliferation, adhesion, migration, and cytotoxicity, allowing dynamic assessment of NM-induced cellular responses

The next frontier in nanotheranostics involves integrating the real-time kinetic data from the RTCA system with advanced imaging. Current studies can leverage the xCELLigence RTCA eSight, which utilises fluorescence microscopy, to visually track and quantify fluorescently labelled NMs and correlate their uptake and localisation with the measured change in the CI. Furthermore, to eliminate the requirement for dyes and labels, a significant future technical upgrade could involve integrating specialised darkfield or hyperspectral imaging optics, potentially allowing the eSight to quantitatively track the label-free internalisation and spatial distribution of nanotheranostics in real-time by detecting the light scattered by the NMs (Figure 3).

Nanocarriers are shown to exploit both passive (EPR effect) and active (receptor-mediated) targeting within the tumor microenvironment, with theranostic and co-delivery platforms enhancing specificity and overcoming resistance. Selective cytotoxicity is evidenced by differential CI suppression in cancer versus normal cells (e.g., HeLa vs. HEK293), while dose-dependent effects of silver nanoparticles on A549 lung cancer cells reveal graded inhibition of proliferation and attachment. Gallium and indium-based NPs induce antiproliferative and apoptotic responses in PC-3 prostate cancer cells, validated by increased Caspase 3/7 activity. Fluorescent carbon fluoroxide NPs (CFO-NPs) further demonstrate selective cytotoxicity across A549 and PC-3 lines. Synergistic and anti-metastatic effects are observed with curcumin-piperine and Thymus vulgaris-loaded gold NPs, suppressing migration in MCF-7 and MDA-MB-231 breast cancer cells. Collectively, this platform enables high-resolution kinetic profiling of NM bioactivity, supporting rational design of targeted nanotherapeutics and mechanistic dissection of cancer cell fate.

### Targeting cellular barrier integrity

The use of the xCELLigence RTCA in assessing the vascular endothelium barrier integrity by inhibiting the glycolytic pathway of cancer cells was mentioned previously (Pawlos et al. 2023). Assessing cell barrier integrity of either endothelial cells surrounding cancer or of cancer cells directly with the xCELLigence RTCA has proven valuable. For example, a study by Schneider et al. (2021) used the system to demonstrate that interleukin-1 beta (IL1B), a pro-inflammatory cytokine, was able to break down the endothelial barrier of a HUVEC monolayer. They also demonstrated that the addition of interleukin-1 receptor antagonist (IL1RA) could prevent this breakdown in a dose-dependent manner, thereby suggesting that IL1RA could play a protective role in maintaining endothelial integrity during tumour progression.

Another critical barrier shown to be the focus in cancer research is the blood-brain barrier for which the xCELLigence RTCA has also been proven useful. Understanding and manipulating the blood-brain barrier is vital for turning currently untreatable brain tumours into diseases that can be effectively managed with systemic therapies. For example, Mokoena et al. (2025) investigated how U87 glioblastoma (GBM) cells affect the blood-brain barrier (BBB) using a brain endothelial cell line (bEnd. 3) as a model. They used two approaches: a conditioned media model to assess the effect of secreted molecules from U87 on bEnd. 3 and a co-culture U87- bEnd.3 models to analyse the impact of direct cell-to-cell interaction. The results showed that while secreted factors increased cell migration via a typical migration scratch assay for the conditioned medium model and a CIM plate for the co-culture model, direct contact between the cells significantly increased barrier permeability. Both models demonstrated an increase in the expression of the tight junction proteins ZO-1 and occludin, indicating that both soluble factors and physical contact from glioblastoma cells can alter the integrity of the BBB model. For the conditioned media model, 1000 bEnd.3 cells were seeded in each well of the DP system. For the co-culture model, bEnd.3 cells were seeded on the bottom chamber while 1200 U87 cells were seeded in each well of the top chamber. In another study by Vézina et al. (2021), the impact of regadenoson, an adenosine A2A receptor agonist, on brain endothelial cell barrier function was investigated. The study found that regadenoson caused a significant but temporary decrease in the cell index of mouse brain endothelial cells, suggesting a transient disruption of the barrier. The barrier function began to recover after about three hours and returned to normal by eight hours. This finding is significant for understanding how drugs could be delivered more effectively across the BBB.

### Exposure studies

The majority of cancer research addresses targeted approaches within controlled conditions. However, research has also shown the detrimental effects of unintended exposure to certain chemicals, potentially leading to favourable conditions for cancer cell growth. For example, a study by Farasani and Darbre (2021) utilised the xCELLigence system to investigate the effects of triclosan, an antimicrobial agent found in household and personal care products, on the migration and invasion of MCF-10 F human breast epithelial cells. At a density of 20,000 cells per well, impedance changes were measured every 15 min over an extended period, revealing triclosan’s impact on cell motility. These kinetic profiles provided insights into the long-term effects of triclosan exposure, suggesting its potential role in promoting cancer progression through enhanced migratory and invasive behaviours.

## Advances in the RTCA system: Use of xCELLigence in complex 3D model systems

The xCELLigence system was originally developed for adherent monolayer cell lines. Cells, tissues, and organs are 3-D structures, and as such, 3-D models preserve the physiological and physical gradient inside cells more effectively (Gao et al. 2021). For example, 2-D cancer cell cultures do not represent cancer expression, whereas cancer cells undergo significant 3D phenotypical changes (Ding et al. 2024). Assessing 3D model systems was not possible until recently with the eSight model. Takahashi et al. (2021) utilised the xCELLigence system to investigate the cytolytic activity of cytotoxic T lymphocytes (CTLs) and NK cells on Fukushima patient-derived organoids (F PDOs). They specifically used an organoid line, RLUN021, which expressed high levels of MICA, a protein known to activate NK cells. The RTCA system successfully monitored the changes in impedance signals as the immune cells killed the organoid cells, demonstrating the feasibility of using this technology to evaluate immune cell activity in a patient-specific context. Shabalina et al. (2021) employed the RTCA system to study cell migration in 3D multicellular tumour spheroids (MCTS) derived from A549 NSCLC cells. Each MCTS, consisting of approximately 200 cells with a radius of 135 ± 10 μm, was cultured on collagen gel or plastic substrates. The system revealed substrate-dependent migration starting 4–16 h post-seeding, with the RTCA method accurately capturing uneven cell migration from the MCTS, providing a precise assessment of migratory dynamics in a 3D tumour microenvironment relevant to NSCLC progression.

Similarly, Madsen et al. (2025) utilised the xCELLigence RTCA system to evaluate CAR-T cell cytotoxicity in advanced 3D heterospheroid models incorporating mCherry/luciferase-labelled cancer cells, fibroblasts, and CD14 + monocytes. Heterospheroids, generated in ultra-low attachment plates, were infiltrated with monocytes before introducing EGFR-CAR T cells, with untransduced T cells (UTDs) as controls. Cytotoxicity against HT-29 spheroids, with or without monocytes, was assessed via a decrease in Total Red Area measured by the xCELLigence system, revealing monocyte-dependent protection against cancer cell killing and highlighting the system’s ability to model complex tumour microenvironments.

## Comparison with conventional endpoint assays

**Figure 4:**
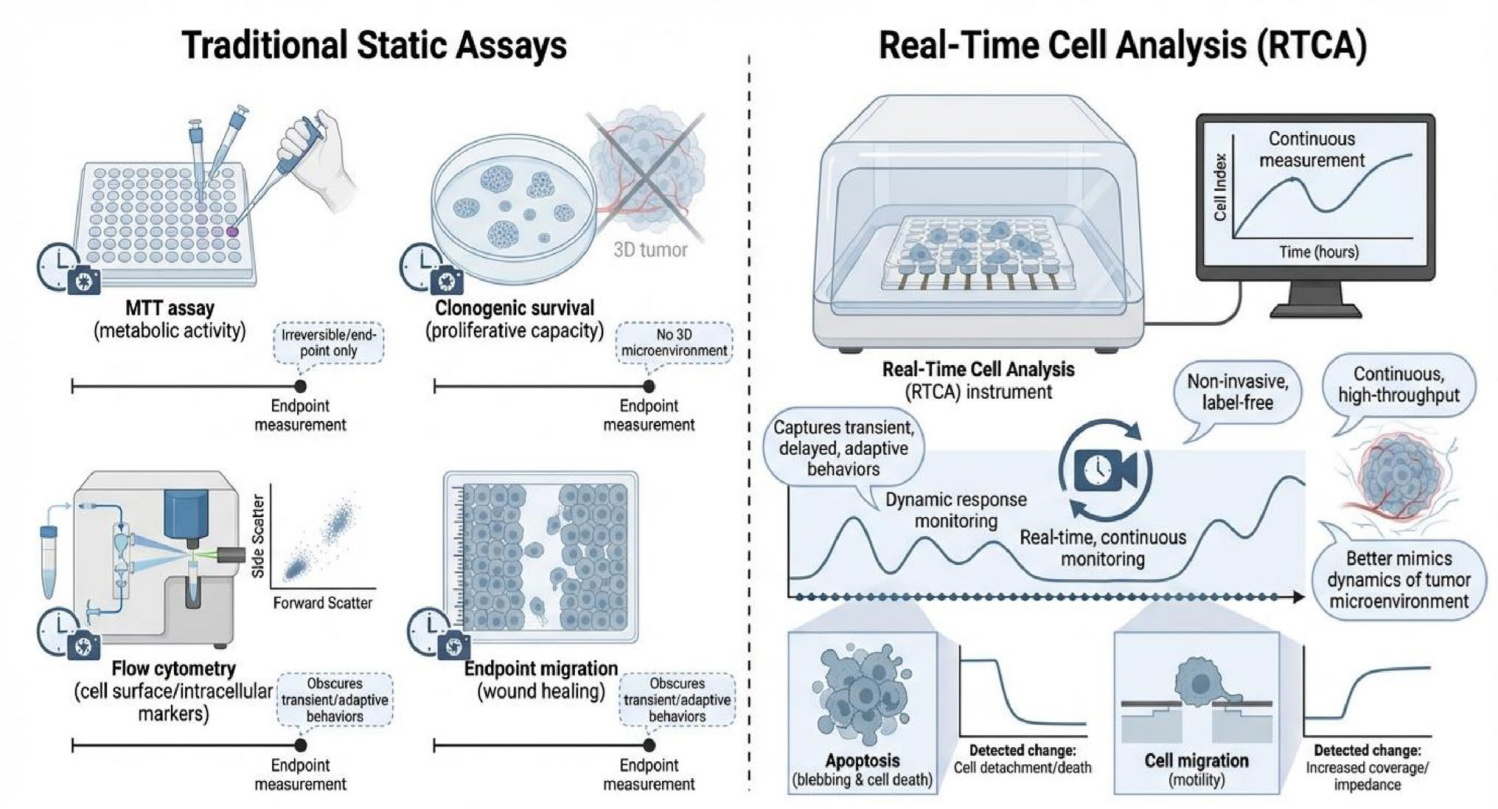
Comparison between traditional static assays and RTCA. Traditional in vitro assays measure cancer cell behavior at singular, fixed time points, missing crucial dynamic and adaptive changes. RTCA provides non-invasive, high throughput, and continuous monitoring enabling researchers to resolve transient, delayed, or adaptive cellular behaviors relevant for understanding drug resistance and tumor dynamics

Conventional endpoint assays such as MTT, XTT, clonogenic survival, and Transwell migration assays remain widely used in cancer research (Pijuan et al. 2019). However, they are inherently limited by their static nature. These assays typically fail to capture transient cytostatic effects, delayed cytotoxicity, or adaptive resistance mechanisms. In contrast, RTCA provides continuous, label-free monitoring that reveals temporal patterns of cellular response. Treatments that initially suppress proliferation but later permit recovery can be readily identified through CI kinetics, whereas endpoint assays may misclassify such responses as uniformly cytotoxic (Kim et al. 2022). Similarly, RTCA-based migration assays eliminate variability associated with staining, manual counting, and endpoint selection, thereby (Figure 4).

improving reproducibility. Nevertheless, RTCA is not without limitations, including reduced applicability to non-adherent cells and potential confounding effects of medium conductivity. A complementary, rather than replacement, role alongside established assays is therefore recommended.

## Translational implications for drug development and precision oncology

The ability of RTCA to capture dynamic cellular responses has significant implications for translational cancer research (Stefanowicz-Hajduk and Ochocka 2020). Time-resolved impedance profiling enables early detection of resistance-associated phenotypes, optimization of dosing schedules, and improved stratification of therapeutic responses in preclinical models. In immuno-oncology, RTCA-based co-culture assays provide a scalable platform for evaluating immune cell potency and tumour susceptibility, supporting the development of predictive immunotherapeutic strategies. Furthermore, integration of RTCA with three-dimensional culture systems, patient-derived cells, and multi-omics analyses holds promise for enhancing the predictive value of preclinical screening (Jose et al. 2024). By bridging mechanistic insight with functional phenotyping, RTCA may contribute to more rational and efficient translation of candidate therapies into clinical evaluation.

## Technical limitations and experimental considerations

Despite the recent advances in cancer research with the help of the xCELLigence RTCA it should be noted that some limitations still exist. First, the technology is restricted primarily to adherent cell models and may not fully capture the behaviour of non-adherent or suspension-based cancer cells without assay adaptation (Kho et al. 2015; Khan et al. 2025). Additionally, impedance signals represent an aggregate measure of cellular behaviour and cannot independently distinguish between specific biological events such as apoptosis, necrosis, or senescence without complementary analyses. Variability in electrode coverage, plate format, and cell type-specific adhesion properties may also influence CI measurements, necessitating careful standardisation across experiments (Alsaç et al. 2025). These limitations underscore the importance of integrating RTCA with complementary biochemical, molecular, and imaging approaches to achieve comprehensive biological interpretation.

## Future perspectives and emerging directions

Future developments in RTCA technology are likely to focus on integration with advanced culture models, including tumor organoids, microfluidic systems, and immune-competent co-cultures. Coupling impedance-derived kinetic data with artificial intelligence–driven pattern recognition and multi-omics profiling may further enhance mechanistic insight and predictive accuracy. Standardization of data analysis pipelines and broader regulatory acceptance will be critical for expanding the role of RTCA in drug discovery and translational oncology.

## Conclusion

The xCELLigence RTCA system is a powerful, label-free technology in cancer research, providing unique kinetic resolution for monitoring cellular processes like proliferation, cytotoxicity, migration, and invasion. Its ability to provide continuous data on drug interventions allows researchers to precisely identify therapeutic windows and the mechanisms of action. Recent advances have further expanded its utility into complex 3D models, such as spheroids and organoids, allowing for the study of immune cell activity and migration in a context more reflective of in vivo tumour microenvironments. While the system faces some limitations, continuous advancements, such as integrating it into complex models and adapting its use for immunotherapy assessments, indicate its value. Moving forward, the xCELLigence RTCA system will remain an indispensable tool for high-throughput screening and detailed functional analysis, driving progress in oncology by offering a dynamic and comprehensive perspective on cellular fate and behaviour.

In conclusion, the xCELLigence RTCA system represents a transformative tool for cancer research by enabling continuous, label-free assessment of cellular behaviour with unprecedented temporal resolution. Its application across diverse therapeutic strategies demonstrates its versatility and capacity to reveal dynamic treatment responses that are often missed by traditional endpoint assays. Future advances that integrate RTCA with high-content imaging, three-dimensional tumour models, and patient-derived systems are likely to further enhance its translational relevance. As cancer research continues to move toward more predictive and personalised experimental models, impedance-based real-time analysis is poised to play an increasingly central role in preclinical oncology.

## Data Availability

No datasets were generated or analysed during the current study.
